# An injury profile of musculoskeletal injuries in CrossFit athletes in KwaZulu-Natal, South Africa

**DOI:** 10.17159/2078-516X/2025/v37i1a18993

**Published:** 2025-06-15

**Authors:** NA Simmons, JD Pillay

**Affiliations:** 1 Department of Chiropractic, Durban University of Technology, Durban, South Africa; 2 Faculty of Health Sciences, Durban University of Technology, Durban, South Africa

**Keywords:** fitness, exercise-related injuries, prevalence, risk factors

## Abstract

**Background:**

CrossFit is a strength and conditioning program identified as a relatively new sport. It has gained widespread popularity since its inception in South Africa in 2007. Consequently, there is growing interest in the types of injuries and injury profiles associated with the sport.

**Objectives:**

This study aimed to determine the prevalence and injury profile of musculoskeletal injuries among CrossFit athletes in KwaZulu-Natal, and to identify associations between the injury profile and selected risk factors, as well as the type of professional care sought following an injury.

**Methods:**

A questionnaire-based study involving 181 CrossFit athletes in KwaZulu-Natal, South Africa, was conducted and included sections on demographics, injury characteristics, and professional care sought. The data were analysed using SPSS Software version 28. Categorical variables and frequency tables summarised the prevalence, selected risk factors, site of injuries and management of musculoskeletal injuries.

**Results:**

Among the 181 participants, 29% (n=53) reported musculoskeletal injuries. The most frequently reported type of injury was a strain/tear (64%, n=34), with the shoulder being the most injured site (38%, n=20). Participants reported moderate pain levels in 49% (n=26) of cases; most injuries (73%, n=39) were caused by weightlifting.

**Conclusion:**

Injuries are relatively common in CrossFit. This study highlights the prevalence, causes, and management of musculoskeletal injuries in a fairly new yet increasingly popular fitness programme. It highlights the need for injury prevention strategies and proper training techniques to ensure the safety and well-being of individuals participating in this sport.

CrossFit is a strength and conditioning program that has gained widespread popularity since its founding by Greg Glasman in 1996 and its inception in 2009.^[1]^ Anecdotal evidence suggests that, in South Africa, the sport was founded in 2007. CrossFit is a type of exercise that incorporates rapid and successive high-intensity movements ^[2]^ and is becoming an increasingly popular fitness option. The program comprises ten fitness categories: cardiovascular/respiratory endurance, stamina, strength, flexibility, power, speed, coordination, agility, balance, and accuracy.^[3]^

According to Glassman,^[1]^ CrossFit aims to better prepare athletes for the unpredictability they will encounter in their daily routines. This is accomplished by engaging in a routine of functional exercises that change quickly and are performed at a high level of intensity.^[1]^ Paine et al.^[4]^ highlights that CrossFit aims to carry out a conditioning workout regimen designed to achieve a generalised and comprehensive fitness level. All participants share a common workout space, known as a “box”, regardless of their fitness level. The head coach of each box designs the workout routines and intensity levels according to their unique approach and experience. Class coaches guide athletes through these workouts, providing extra support when necessary. However, adaptations and how assistance is implemented can vary, leading to differences in individual workouts despite the shared principles. These variations may be influenced by the athlete’s perceived skill level, the specific box they attend and the head coach or class coach leading the session.

While CrossFit has many advantages, such as increased flexibility, strength, and cardiovascular fitness, concerns have been raised about its resultant risks of musculoskeletal injuries.^[2]^ This may be due to several factors, including high-intensity workouts. The demanding nature of CrossFit training can place considerable stress on the body, thereby increasing the risk of overuse injuries alongside the complexity of movements. Many CrossFit exercises involve complex movements that require precise technique and coordination, which can lead to injuries if not performed correctly. Additionally, CrossFit often emphasises rapid progression, which can place undue strain on the body if it is not complemented by adequate rest and recovery. Furthermore, the quality of coaching can vary, leading to improper form and an increased risk of injury. Each person’s body is unique therefore, training regimens that work for one individual may not be suitable for others.^[5]^

As CrossFit is relatively new on the sporting arena, only a few studies have been conducted to examine the sport, with particular reference to injuries.^[6]^ Weisenthal et al.^[7]^ found an overall injury rate of 19% in a survey of 386 adult athletes.^[7]^ More recently, de Queiroz Szeles et al.^[8]^ summarised the prevalence of Cross-Fit-related musculoskeletal injury ranging from 19% to 74% in periods ranging from 6 to 18 months, and an incidence ranging from 2.1 to 3.1 injuries per 1000 hours of exposure.^[8]^

Studies have emphasised the crucial role of utilising proper technique, suitable programming, and progressive advancement to reduce the risk of injury.^[7]^ Further studies are warranted that concentrate on the injury patterns and the connections between injury profiles and specific risk factors. Investigating the injury profile of CrossFit athletes is essential to fully understand the challenges and opportunities associated with this fitness regimen. It also allows for the development of tailored interventions aimed at reducing and managing injuries, including training protocols and athlete education.

Therefore, the study aimed to determine the prevalence and injury profile of musculoskeletal injuries among CrossFit athletes, including the location, nature, and severity of these injuries. Additionally, the study sought to identify any associations between the injury profile and selected risk factors for injury, such as age, gender, height, weight, and ethnicity. Finally, the type of care and management that CrossFit athletes pursued for various types of injuries was investigated

## Methods

### Study design

A questionnaire-based cross-sectional approach was used to gather information from CrossFit athletes regarding their level of activity in the sport and the injuries sustained while participating in it.

### Study population

The study population included CrossFit athletes in KwaZulu-Natal, South Africa. The population was determined by the number of members of the CrossFit-affiliated gyms willing to participate in the study. Healthy adults between the age of 18–40 who participated in CrossFit at least twice a week were invited to take part in the study.

### Sample size

The estimated total number of CrossFit athletes at the affiliated boxes in the eThekwini region was 340. Based on a population of 340 and an estimated population prevalence of CrossFit injuries of 50% with 5% precision^[8]^ (half-width of the 95% confidence interval), this study aimed to recruit 181 participants.

### Measurement tool

The questionnaire was created by reviewing similar studies with similar surveys,^[9, 10]^ and was modified and adapted to fit the study objectives. The questionnaire comprised four sections: Section A–demographics; Section B–participant characteristics; Section C–characteristics of injuries sustained, and Section D–additional activities. Both sections B and C included general questions about the nature of injury, duration, sporting time lost, working time lost and if there was permanent damage that resulted.

An expert focus group reviewed the questionnaire. This ensured an organised discussion and examination to enhance the internal validity of the questionnaire. The focus group comprised a student researcher, a research supervisor, a chiropractic staff member, a chiropractor with relevant research experience, and two participants who met the study’s inclusion criteria. Following this, changes were made to the questionnaire, resulting in a modified version used in the study.

### Ethical considerations

The study received ethical clearance from the institutional research ethics committee (IREC 178/23) at Durban University of Technology. The gym owners gave permission to conduct research within CrossFit-affiliated gyms. The CrossFit athletes who participated in the study received letters explaining the research and assuring their data were kept confidential. Each participant signed a letter of informed consent before participating.

### Statistical analysis

The data were analysed using IBM SPSS Version 28. Categorical variables were summarised using frequencies and percentages. A chi-square goodness-of-fit test was applied to single categorical variables measuring different aspects of injuries sustained to determine a significant selection of responses. The binomial test was used to determine whether different healthcare workers were significantly consulted or not. The differences in injury frequency across age and BMI were examined using ANOVA; Fisher’s exact test explored the relationship between skill levels and incidence of injury. Finally, Spearman’s correlation was used to determine the correlation between injury incidence and years doing CrossFit.

## Results

### Participants’ characteristics

A total of 186 questionnaires were completed. However, five were incomplete and therefore not included in the analysis. Thus, 181 questionnaires were suitable for analysis, resulting in a completion rate of 97%.

Approximately half (51%, n=92) of the participants identified as female and 49% (n=88) as male, with one participant identifying as Other.

The mean age was 30.0±5.6 years, and the mean body weight was 78.6±15.8 kg. Their heights ranged from 1.56m to a maximum of 1.98 m, with a mean of 1.73m±9.4 cm.

### Training history and CrossFit participation

Most respondents (64%, n=116) preferred weightlifting as their primary form of exercise, while 21%, n=38) of participants favoured bodyweight exercises. Even less common, 11% (n=19) of respondents selected running as their preferred discipline, with a preference for swimming indicated by 3.3% (n=6) of participants. The study revealed that most participants (78%, n=142) typically spent between 30 minutes and 1 hour on their CrossFit training sessions. Additionally, of the total participants, 77% (n=140) reported that they usually stretched before training, while only 40% (n=72) stretched after training.

Skill level was divided into four categories: beginner (n=34), amateur (n=56), intermediate (n=74) and advanced (n=17). According to the data, beginners were less prone to injuries with 94% (n=32) not reporting any injury. Advanced individuals were the least prone to injury, with 41% (n=7) experiencing only one injury and 47% (n=8) reporting no injury. In contrast, participants with an intermediate skill level were the most vulnerable to injury (18%, n=13).

### Injuries sustained

A significant proportion of respondents reported sustaining injuries to their shoulder (38%) or lumbar back (28%). The injuries were diagnosed as strains or tears (64%) and occurred while weightlifting (74%). Their pain levels were moderate (49%), and the injuries lasted for at least two weeks (85%).

### Professional healthcare in managing musculoskeletal injuries among CrossFit athletes

Injured CrossFit athletes often seek treatment from various healthcare professionals and undergo a wide range of treatments, which can vary significantly (Table 1).

Of those who reported experiencing injuries, 58% (n=28) of participants sought the advice of a chiropractor, making this the most consulted practitioner. Physiotherapists were also consulted frequently (56%, n=27). Occupational Therapists and General Practitioners were the least consulted professionals (2%, n=1), respectively (Table 2).

Acupuncture and dry needling were the most commonly utilised forms of treatment, with 71% (n=34) of patients choosing this method. Chiropractic adjustments ranked as the second most popular treatment option (54%, n=26). In contrast, steroidal injections had the lowest uptake, with 96% (n=46) of participants not selecting this treatment option (Table 2).

### The association between injuries, general characteristics and training history

Of the 91 females surveyed, injuries were self-reported as follows: 69% (n=63) indicated no injuries, 20% (n=18) indicated one injury, and 11% (n=10) indicated multiple injuries.

Similarly, of the 88 male participants, 72% (n=63) reported no injuries, 18% (n=16) reported one injury, and 10% (n=19) reported multiple injuries.

Analysis showed a significant relationship between age and CrossFit injuries, with the average age varying significantly based on the number of injuries sustained (F(_2, 177_)=5.201, p=0.006). Further analysis using Tukey’s *post hoc* test revealed that individuals who had experienced one injury (n=34, mean age=31.9±5.7 years) were older than those who had not had an injury (n=127, mean age=29.1±5.4 years), p=0.022.

There was no significant correlation between BMI and the frequency of injuries. However, a significant relationship was demonstrated between skill levels and the number of injuries sustained (Fisher’s=22.5, p=0.001). The results showed that 94% of the beginners did not sustain injuries. In comparison, a significant proportion (18%) of those with intermediate-level skills sustained multiple injuries, and a significant 41% of those with advanced skills sustained one injury. Furthermore, there was a moderate positive correlation between the number of injuries sustained and the total years of doing CrossFit (*rho*=0.40, p<0.001). A weak positive correlation was found between the number of injuries sustained and the frequency of CrossFit sessions per week (*rho*=0.24, p=0.002).

## Discussion

A total of 29% (n=53) of CrossFit athletes reported sustaining injuries. This finding aligns closely with the 31% and 30% rates reported by Feito et al.^[6]^ and Sprey et al.^[10]^, respectively. Nevertheless, our findings are higher than those reported by Weisenthal et al.^[7]^ and Montalvo et al.^[11]^, which were 19% and 26%, respectively. Our results also suggest that the risk of injury associated with CrossFit is comparable to that of other demanding forms of exercise, including weightlifting, running (short, middle, and long distance), and triathlon, as noted by Spray et al. (2016).^[10]^ The main distinction between our study and others is that our questionnaire did not restrict reporting to injuries within a specific timeframe. In contrast, most other studies typically focused on a period prevalence of 12 months.

### Site of injury

The area most commonly affected was the shoulder (38%), followed by the lower back (28%) and knee (11%). These findings are consistent with prior injury profile studies.^[2,7,11–12]^ Montalvo et al.^[11]^ identified shoulder injuries as the most prevalent (23%) injury among those who practice CrossFit, followed by knee (16%) and lower back (13%) injuries. These results are consistent with other CrossFit literature, which shows a high frequency of shoulder, lower back and knee injuries.^[3,7]^ The prevalence of pain and injury among CrossFit athletes warrants further studies, as the high intensity, high repetition, complex techniques, and heavy weights used in CrossFit may contribute to developing joint/muscle pain and injury.^[2]^

The literature on CrossFit describes the knee as a common area of complaint.^[2,7]^ Knee complaints may be partly due to heavy weights that stress joint structures significantly. ^[13]^ Ballistic-type lifts, like the snatch and clean and jerk, focus on the rapid movement of heavy weights, placing stress on the soft tissue structures and leading to strains, thus increasing the risk of injuries.^[13]^ The knee is also a common region of injury in endurance sports such as running.^[14–16]^ The combination of lifting weights in a ballistic style and engaging in endurance running in CrossFit may increase the risk of injury for athletes. Possible reasons for these areas commonly sustaining injuries may be due to the structures being placed under high levels of stress at a high frequency and repetition. Many overhead exercises are performed during CrossFit, often at high loads and have many technical aspects to the movements. Therefore, the structures in the shoulder are subjected to strain as a repetitive hyper-flexed, abducted, and internally rotated position is adopted. The structures eventually fatigue, resulting in a lack of technique and form, which may result in injury.^[3]^ Athletes must be aware of common risk areas to prevent injuries during training and prioritise maintaining proper form.^[7]^

### Type of injury

The most common injury type during CrossFit was muscle strains or tears, accounting for 64% (n=34) of the injury cases reported. The high emphasis on soft tissue structures leads to a greater risk of strains, as highlighted in a previous study.^[15]^ CrossFit’s high-intensity workouts, complex movements and potential for rapid progression can increase the risk of muscle strains.^[2,17]^ Inadequate warm-up and cool-down routines, combined with individual factors like age and fitness level, can further exacerbate this risk.^[2,3,17]^

Regarding the severity of injuries in CrossFit athletes, most injuries were acute (up to 3 months) and required medical attention (79%, n=48). Hak et al.^[2]^ also reported that most injuries were acute; however, they found that most injuries were mild to moderate and often did not require medical attention, leading most athletes to apply various self-care approaches. In contrast, Weisenthal et al.^[7]^ found that 74% of CrossFit athletes reported injuries severe enough to prevent them from training. This was further supported by the results of the present study, which showed that 83% (n=44) of athletes were unable to train due to their injuries. These findings highlight the importance of proper technique, sufficient rest, and listening to one’s body to minimise the risk of injuries in CrossFit.

### Risk factors

Skill level was divided into four categories: beginner, amateur, intermediate, and advanced. Our findings revealed that those categorised as advanced CrossFit athletes were less prone to injuries at only 9% (n=17). The study by Montalvo et al.^[11]^ suggests that athletes who dedicate more time to training are less likely to suffer from injuries, possibly due to the gradual improvement of their skills and techniques. Conversely, intermediates were found to be the most susceptible to injuries, with a prevalence rate of 41% (n=74). In a study conducted by Sprey et al.^[10]^ injury rates were examined in relation to athletes’ profiles, training routines, and sports history. The findings revealed that those who practised CrossFit for more than 6 months showed significantly (p=0.004) higher injury rates than those who practised for less than 6 months. These findings demonstrate a clear relationship between skill level and injury risk in CrossFit. Advanced athletes, with more experience and training time, are less prone to injuries. Conversely, intermediate-level athletes may be pushing their limits without adequate skill development and are at a higher risk of sustaining injuries. These findings highlight the importance of gradual progression, proper technique, and continuous skill development to minimise the risk of injuries in CrossFit.

Body mass index was not a risk factor for injury. This finding aligns with those of Montalvo et al.^[11]^, but it may be due to the limited sample size in both studies.

No significant correlation was found when comparing the monthly duration of CrossFit practice to injury rates among athletes. However, a previous study^[10]^ found that athletes who had been practising CrossFit for more than six months and were training for competitions were more likely to sustain injuries. In contrast, Montalvo et al.^[11]^ found that a longer duration of CrossFit reduced the rate of injury, which contradicts the former study. It is unclear whether a longer training period decreases injury rates due to an increased skill level or increases the risk for injury due to athletes attempting more advanced techniques and intense workout programs. Since there are conflicting studies with different outcomes, it is difficult to draw any conclusions, especially since the current study found no correlation between duration and injury rate. Therefore, more finite research is needed to accurately determine which factors influence injury in CrossFit athletes, specifically around the duration of their participation in the sport.

### Professional management of injury

Our data revealed that only 2% (n=1) of patients visited a general practitioner. In comparison, most patients (57%, n=27) opted for non-invasive care, such as chiropractic and physiotherapy. A study by Giles and Muller,^[18]^ showed that the highest proportion of early recovery was observed in chiropractic manipulation (27%), followed by acupuncture (9%) and medication (5%).^[21]^ The study concluded that manipulation achieved the best overall results with improvements. However, it emphasised that the treatment for chronic spinal pain should not rely solely on manipulation, acupuncture or nonsteroidal anti-inflammatory drugs but rather on a combination of these treatments. According to a study by El-Tallawy et al.,^[19]^ patients are increasingly choosing non-invasive care options for musculoskeletal pain and injury instead of pharmacological or surgical treatment. Furthermore, in a more recent study by Skelly et al., ^[20]^, the efficacy of nonpharmacological therapies in addressing specific chronic pain conditions was highlighted.^[20]^ These included exercise, rehabilitation, acupuncture, and mind-body practices, demonstrating notable improvements in function and pain reduction.^[20]^

Our findings similarly suggest that a multidisciplinary approach, incorporating exercise, rehabilitation, acupuncture and mind-body practices, to name a few, can effectively manage specific chronic pain conditions, leading to lasting improvements in function and pain reduction. Future research should focus on comparing the effectiveness of these interventions across different chronic pain conditions and evaluating their long-term sustainability.

### Key findings

- The most injured areas in CrossFit athletes were the shoulder (38%), lower back (28%), and knee (11%).
- Injury rates for CrossFit athletes were below 30%, similar to those in related sports.
- If injuries occurred, they were mostly considered acute injuries.
- Risk factors such as age and skill level increased the risk of injury prevalence in CrossFit athletes.
- When injured, athletes sought treatment, preferring chiropractors and physiotherapists over general practitioners for musculoskeletal injuries.
- Acupuncture and dry needling were the most used form of treatment types.

### Strengths of the study

This study represents the first of its kind in Kwazulu-Natal, South Africa, focusing on the epidemiology of musculoskeletal injuries in CrossFit athletes. While previous South African studies^[21]^ focused on the specific activities and events of the CrossFit games, this study specifically directs its attention to the training aspects at CrossFit-affiliated gyms in South Africa, rather than the CrossFit games. Another notable strength of this study is the added knowledge about musculoskeletal injuries in CrossFit athletes, which holds value for healthcare professionals in managing injuries related to this sport.

### Limitations and recommendations

To participate in the study, individuals had to be current members of a Cross Fit-affiliated gym. Athletes who stopped due to injury were not included in the study, and their information was lost. Additionally, the study had a minimum age requirement of 18 years. This meant that younger CrossFit athletes could not participate in the study, which may have excluded a population of athletes more prone to injury due to various age-related factors. In future studies, it is crucial to expand the sample size by incorporating athletes younger than 18 years old and those with a history of injuries who are currently inactive. This can be achieved through an online survey that will reach more individuals who do not currently attend the CrossFit-affiliated gym, enabling the survey to have a broader scope reach. Furthermore, the self-reported nature of the study and its cross-sectional design pose some limitations to the accuracy of findings. We would therefore recommend a longitudinal study of at least 6 months to obtain more accurate information on injuries not limited to recall. Due to the limited literature on musculoskeletal injuries in CrossFit athletes, the study compares to other sports, such as weightlifting. While this approach helped identify similarities between the sports, comparing the data to studies that focus solely on CrossFit would be more beneficial.

## Conclusion

The findings of this study suggest that injuries are a relatively common occurrence in the sport. The most frequently reported type of injury was a strain or tear, with the shoulder being the most commonly affected area. Participants reported moderate pain levels in 49% (n=26) of cases, indicating that injuries were not severe enough to result in significant discomfort. Most injuries (73%, n=39) were caused by weightlifting, highlighting the importance of proper technique and safety measures when performing this type of exercise.

To reduce the risk of musculoskeletal injuries in CrossFit, athletes should prioritise proper warm-up, gradual progression, listening to their bodies, maintaining correct form, adequate rest, and cross-training. Coaches should tailor workouts to individual athletes, provide clear instructions, emphasise technique, educate athletes about injury prevention and encourage rest. By implementing these recommendations, CrossFit athletes can enjoy the benefits of the sport while minimising the risk of injuries and ensuring safety and overall well-being.

## Figures and Tables

**Table 1:** Overview of the injuries sustained by the CrossFit athletes

Indicator	Indicator categories	Frequency (n)	Percent (%)	χ^2^	df	p-value
**Injury location**	Shoulder	20	38	39.3	6	<0.001
	Lumbar back	15	28			
	Knee	6	11			
	Foot/Ankle	4	8			
	Hip/Thigh	3	6			
	Hand/Wrist	3	6			
	Elbow	2	4			
**Diagnosis**	Strain/Tear	34	64	67.1	4	<0.001
	Sprain	6	11			
	Bruise/Wound	4	8			
	Fracture	1	2			
	Other	8	15			
**How it was sustained**	Weightlifting	39	74	124.9	5	<0.001
	Ballistic movements	4	8			
	Cardiovascular training	2	4			
	Post workout	2	4			
	Body resistance training	1	2			
	Other	5	9			
**Pain level**	Very mild	4	8	32.0	4	<0.001
	Mild	10	19			
	Moderate	26	49			
	Severe	10	19			
	Very severe	3	6			
**Duration of injury**	<48 hrs	4	8	14.3	4	0.006
	few days - 1 week	4	8			
	2–4 weeks	17	32			
	1–3 months	14	26			
**Training interruption**	Not at all	9	17	5.538	3	0.136
	Up to 1 month	19	36			
	Up to 3 months	15	28			
	> 3 months	9	17			

χ^2^, chi-square; df, degrees of freedom; n, number of participants

**Table 2:** Treatment sought out and performed on injured CrossFit athletes (n=48)

		Frequency (n (%))		p-value

**Treatment sought**	Chiropractor	**Yes**	**No**	0.312
	
	Physiotherapist	28 (58)	20 (42)	0.471
	
	Biokineticist	27 (56)	21 (44)	0.013
	
	Home remedy	15 (31)	33 (69)	<0.001
	
	General Practitioner	3 (6)	45 (94)	<0.001
	
	Occupational Therapist	1 (2)	47 (98)	<0.001
	
	Other	1 (2)	47 (98)	<0.001

**Treatment performed**	Acupuncture/dry needling	34 (71)	14 (29)	0.006

	Therapeutic massage	29 (60)	19 (40)	0.193

	Chiropractic adjustment	26 (54)	22 (46)	0.665

	Pain medication	18 (38)	30 (62)	0.111

	Rest, Ice, Compress, Elevate (RICE)	18 (38)	30 (62)	0.111

	Steroid injections	2 (4)	46 (96)	<0.001

	Other	41 (85)	7 (15)	<0.001

n, number of participants
